# Case report: Diagnosis and emergency surgery on a young patient with extensive aortic dissection without any risk factors

**DOI:** 10.1186/s12872-021-02216-x

**Published:** 2021-08-26

**Authors:** Masoud Shafiee, Mohsen Shafiee, Noorollah Tahery, Omid Azadbakht, Zeinab Nassari, Reza Baghbani

**Affiliations:** 1 Department of Cardiac Surgery, Abu Ali Sina Hospital, Shiraz University of Medical Sciences, Shiraz, Iran; 2 Department of Nursing, Abadan University of Medical Sciences, Abadan, Iran; 3 Radiology Technology Department, Behbahan Faculty of Medical Sciences, Behbahan, Iran; 4 Department of Medical Emergency, School of Allied Medical Sciences, Bushehr University of Medical Sciences, Bushehr, Iran

**Keywords:** Case report, Aortic dissection, Cardio surgery, Symptoms, Risk factor

## Abstract

**Background:**

Type A aortic dissection is a very dangerous, fatal, and emergency condition for surgery. Acute aortic dissection is a rare condition, such that many patients will not survive without reconstructive surgery.

**Case presentation:**

We present a case 24-year-old male who came with symptoms of shortness of breath and cough. The patient underwent ECG, chest radiology, and ultrasound, where the patient was found to have right pleural effusion while his ECG was normal. In the history taken from the patient, he had no underlying disease, no history of heart diseases in his family. For a better diagnosis, ETT and aortic CT angiography was performed on the patient which confirmed the evidence of dissection. Immediately after the diagnosis, necessary arrangements were made for open heart surgery and the patient was prepared for surgery. The patient was admitted in the cardiac surgery ICU for 5 days and his medication was carefully administered. After the conditions were stabilized, the patient was transferred to the post-cardiac surgery ICU ward. The patient was discharged from the hospital one week after the surgery and returned to the office as an OPD one week after his discharge.

**Conclusion:**

Various risk factors can play a role in creating aortic dissection. Therefore, it is necessary to pay attention to patients’ history for achieving a quick and definitive diagnosis. Therefore, to control the complications of placing the cannula as well as the duration of the surgery, it is very important to reduce the duration of pumping on the patient and to be very careful during the cannula placement.

## Background

**Fig. 1:**
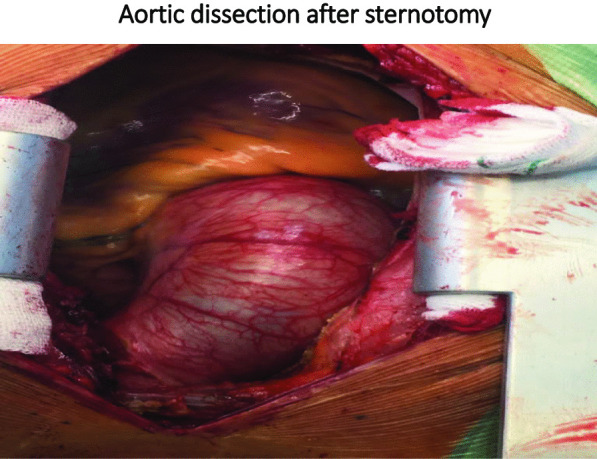
Aortic dissection after sternotomy

Type A aortic dissection is a very dangerous and fatal condition [1] and is an emergency indication for surgery, such that many patients will not survive without reconstructive surgery [2]. Acute aortic dissection is a rare condition with a prevalence of 3 to 4 individuals per 100,000. This condition is very serious since its mortality rate without surgery is 90%, while the mortality rate after surgery and hospitalization is 10–20% [3]. In general, there are many causes for aneurysms, including inherited, degenerative, atherosclerotic, inflammatory, and traumatic causes. Among these reasons, the most important factor is heredity [4]. The aorta has two models: model A, which mostly involves the aortic root, ascending aorta, and aortic arch, and the branches protruding from the aortic arch, and model B, which mostly involves the descending aorta [5]. The etiology of this disease in the thoracic aorta is changes in connective tissues, as well as mechanical, chemical, and genetic changes, but in the abdominal aorta, it is mostly due to inflammatory conditions. Genetics is responsible for 15% of the cases of this disease (Table 1). Hypertension (in two-thirds of patients due to stress on the vascular walls), Turner syndrome, Marfan syndrome, Ehlers-Danlos syndrome, and aortic inflammation (affected by the integrity of the vascular wall) are among the common causes of this disease. In any case, the incidence of symptoms such as chest pain, which starts suddenly [6] and spreads to the back, requires immediate treatment [2]. Many patients have suffered from chronic hypertension, nausea and severe anxiety, focal neurologic symptoms, coma, syncope (in 9% of cases), and pulse deficit. Other symptoms include cardiac tamponade with or without aortic rupture, pseudo-hypotension/peripheral hypotension with central norm tension, the murmur of aortic regurgitation, and acute myocardial infarction [6]. The cause of death in pre- and post-surgery conditions is impaired cardiac perfusion due to the nature of the disease [7]. Open-heart surgery is a well-known and common treatment for large aneurysms [8]. In this study, we reported a case that had no risk factors for aortic aneurysm. However, the delay in diagnosing the disease and the lack of treatment for this patient led to his death (Fig. 1).

**Table 1:**
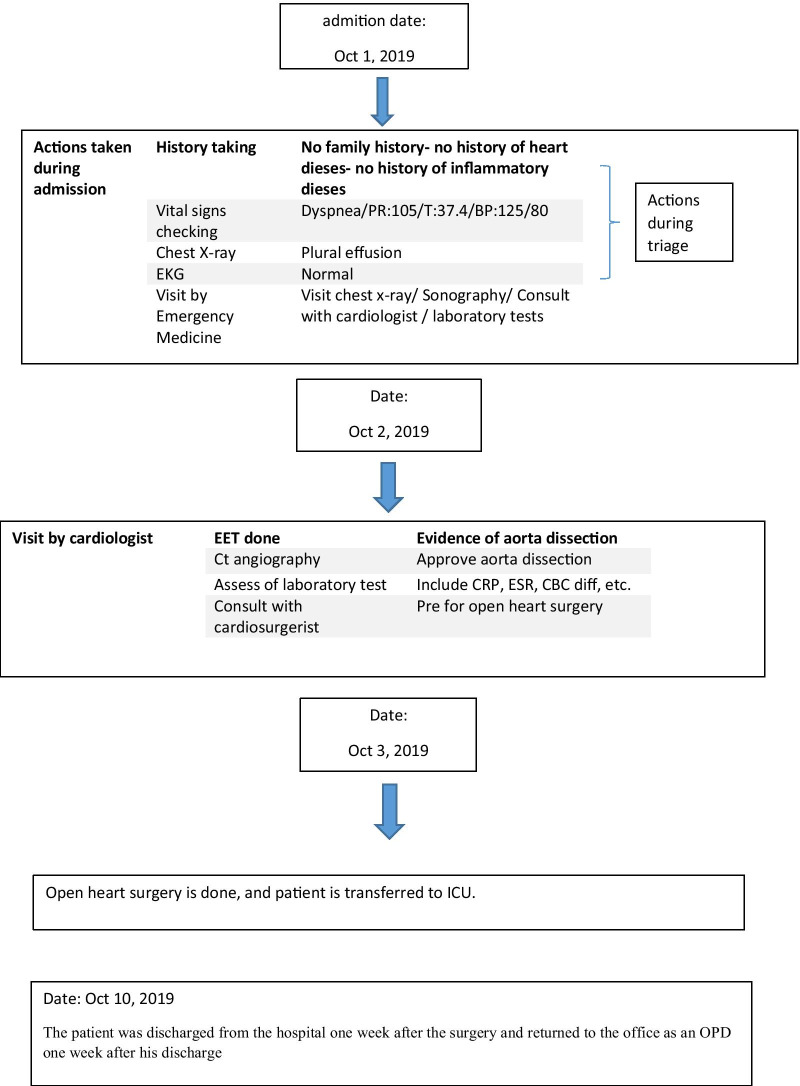
Diagram of the process of diagnosis and treatment of the studied patient

## Case presentation

**Fig. 2:**
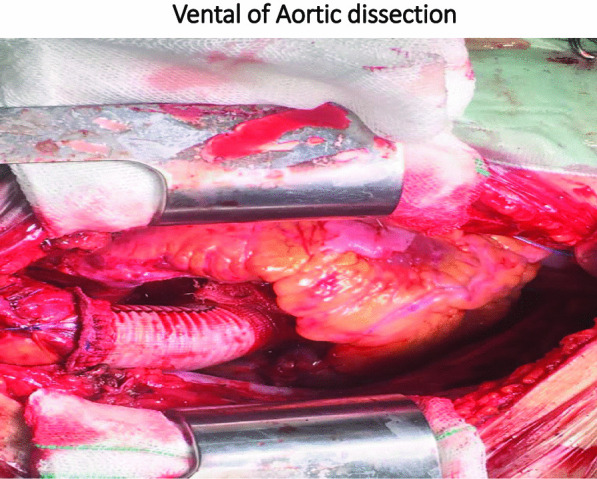
Vental of Aortic dissection

The patient was a 24-year-old male who came with symptoms of shortness of breath and cough. The patient underwent ECG, chest radiology, and ultrasound and was found to have right pleural effusion while his ECG was normal. Early vital signs of the patient at the time of visiting the hospital were BP: 125/80, PR: 105, T: 37.4, RR: 32, SpO_2_ = 96%. The history taken from the patient revealed that he had no underlying disease and no history of heart diseases in his family. Moreover, there was no history of inflammatory diseases in the patient or his family. The patient’s BMI was within the normal range, and he had a history of working in harsh conditions under high pressure. After admission, the patient underwent echocardiography ordered by a cardiologist, where according to the physician, evidence of aortic dissection was observed. For a better diagnosis, echocardiography through the esophagus was performed on the patient which confirmed the evidence of dissection. Since aortic dissection is a life-threatening emergency, an urgent aortic CT angiography was requested and performed as ordered by the patient’s physician, and aortic dissection of type A was confirmed in the patient. Immediately after the diagnosis, necessary arrangements were made for open-heart surgery and the patient was prepared for surgery. Blood samples were sent to a laboratory for the required tests (i.e., antibody, CBC, and similar tests), and to diagnose inflammatory processes and related syndromes tests such as RF, CRP, and IgG, among others, were used. The patient was immediately taken to the operating room for urgent surgery after obtaining informed consent from him and his family. It should be noted that blood samples were taken by professional nurses and sent to the laboratory under special conditions to reduce the error rate. To establish a cardiopulmonary pump for the patient, a number 19 canola was applied and the right femoral artery was used to cannulate the patient. After implanting a cannula in the femoral artery, the patient underwent sternotomy, his pericardium was opened, and his right atrium was cannulated. Then the patient went on the pump and his pump timing started. After performing sternotomy on the patient, we found that the dissection started from the aortic annulus and continued in the ascending aorta before the aortic arch. The size of the false lumen in the aneurysm was about 7–8 cm and led to severe aortic valve failure. After the start of the cardiopulmonary pump, the ascending aortic dissection was opened and imported into the right coronary artery and left main artery through a direct cannula and the patient received cardioplegia. The aortic annulus, ascending aorta, and aortic valve were completely removed. The composite graft with metal aortic valve was number 23 which was anastomosed to the aortic annulus, and the right coronary artery and left main artery were anastomosed to the composite graft. The distal composite was also anastomosed to the aortic arch. Three number 32 chest tubes were embedded in the mediastinum area and the right and left pleura. The patient had no arrhythmias during the surgery. The surgery lasted for about 6 h and the patient’s cardiopulmonary pumping took about 200 min. It should be noted that the patient received two units of blood during the surgery. After surgery, the patient was transferred to the cardiac surgery ICU with complete cardiac and respiratory monitoring and was placed under respiratory monitoring using a ventilator and a hemodynamic monitor upon arrival. The patient underwent intensive care in the cardiac surgery ICU and was constantly monitored for acute post-surgery side effects such as bleeding and cardiac tamponade. During the first 12 h of admission to the cardiac surgery ward, the patient’s mean arterial pressure was maintained between 65 and 70 mm Hg to prevent bleeding, and his systolic pressure was between 80 and 90 mm Hg. Moreover, due to the long duration of the surgery and the high possibility of bleeding, the patient was anesthetized for about 8 h using sedative drugs to ensure the post-surgery conditions. It is worth mentioning that during these 8 h, the patient’s pupils had normal reactions to light and were of normal size. The patient was constantly monitored. ABG was monitored and adjusted every hour. The patient’s electrolyte levels were also corrected. In the ward, the patient underwent chest radiography wherein no evidence of bleeding, tamponade, and pneumothorax was observed. The patient was hospitalized and remained under medical care in the cardiac surgery ICU for five days and his medication was carefully administered. After his conditions were stabilized, the patient was transferred to the post-cardiac surgery ICU ward. The patient was discharged from the hospital one week after the surgery and returned to the outpatient department (OPD) one week after his discharge. After visiting the OPD, the patient underwent CT angiography and echocardiography and his condition was stable. Moreover, his aortic valve, the ascending aorta, and right and left main coronaries worked well and the patient’s heart contraction was satisfactory with EF: 55% (Fig. 2).

## Discussion

In this study, we identified a case with aortic dissection that had no risk factors for this condition other than his gender. In this case, which, unlike other studies, had visited the hospital with respiratory symptoms, pleural effusion was reported [6]. On the other hand, no abnormal changes were observed in the patient’s EKG. In other words, the patient did not have any clear symptoms other than chest pain, which made the diagnosis of the disease by physicians considerably difficult. In this case, CT angiography was used for a definitive diagnosis. Some studies have suggested that bacteria such as *C. septicum* may play a role in the formation of aortic dissection [9]; however, there were no complaints regarding the existence of infectious symptoms by the patient and his WBC differential was normal. Genetics is involved in 15% of the cases of this disease. Common causes of this disease include hypertension (in two-thirds of the patients due to imposing stress on vascular walls), Turner syndrome, Marfan syndrome, Ehlers-Danlos syndrome, and aortic inflammation, which can affect the integrity of the vascular walls [2]. However, contrary to the results obtained from the literature, the patient did not suffer from any genetic disorders, hypertension, or any syndromes. The following are several other predictors of aortic dissection A: The prevalence of this disease is higher among men than women [5]; the prevalence of the disease increases with the increase of age; the risk of the disease increases with the increase of BMI; smoking and alcohol consumption increase the prevalence of the disease. On the other hand, in addition to factors such as sepsis, increased WBC, and INR changes, history of aneurysms can also be effective in causing an aneurysm. However, the studied patient had a normal BMI, along with normal laboratory results. Furthermore, the patient did not have a history of smoking and alcohol consumption and was young, and aortic dissection is rarely observed in this age group [10]. The only risk factors observed in this patient were his gender and working under harsh conditions. As stated in the literature, the prevalence of this disease is higher in men than women [5], and our case study was male; therefore, the existence of this disease in his body is justified. On the other hand, the results of the study by Aparci et al. suggested that environmental and occupational factors can affect aortic aneurysm; thus, this condition is considered an occupational disease. In addition, Aparci concluded that the nature of some occupations, such as sports occupations, heavy and stressful jobs, and military jobs, among others, affects the development of this disease [10]. The medical history of our case revealed that he had worked in very bad weather conditions, under high stress levels, and had carried heavy equipment, which were all occupational factors affecting this disease, consistent with the results of the study by Aparci. Moreover, aortic dissection has many side effects; one of these adverse effects is acute aortic valve failure, which appeared in our patient.

## Conclusion

Type A aortic aneurysm is a dangerous and rare condition, which requires emergency medical treatment**.** Aortic dissection can lead to aortic valve failures, and thus requires immediate action to reduce the complications and mortality rate. Early diagnosis of this disease plays an essential role in its treatment**.** Various risk factors can play a role in the development of aortic dissection. These factors lead to various and atypical symptoms, which makes the disease difficult to diagnose. Therefore, it is necessary to pay attention to patients’ history to achieve a quick and definitive diagnosis. The most definitive method for diagnosing aortic dissection is CT angiography, and the best treatment for an acute aortic aneurysm is heart surgery. Moreover, the effects of gender and working conditions should not be neglected and must be further investigated.

## Data Availability

The datasets used and/or analyzed during the current study available from the corresponding author on reasonable request.

## Abbreviations

AAA: Acute aortic dissection
OPD: Outpatient department
CT scan: Computer topography
EKG: Electrocardiogram
BMI: Body Mass Index
INR: International normalized ratio
WBC: White blood cell
